# Immunopotentiation of Different Adjuvants on Humoral and Cellular Immune Responses Induced by HA1-2 Subunit Vaccines of H7N9 Influenza in Mice

**DOI:** 10.1371/journal.pone.0150678

**Published:** 2016-03-01

**Authors:** Li Song, Dan Xiong, Maozhi Hu, Xilong Kang, Zhiming Pan, Xinan Jiao

**Affiliations:** 1 Jiangsu Co-innovation Center for Prevention and Control of Important Animal Infectious Diseases and Zoonoses, Yangzhou, Jiangsu 225009, China; 2 Jiangsu Key Laboratory of Zoonosis, Yangzhou University, Yangzhou, Jiangsu 225009, China; Instituto Butantan, BRAZIL

## Abstract

In spring 2013, human infections with a novel avian influenza A (H7N9) virus were reported in China. The number of cases has increased with over 200 mortalities reported to date. However, there is currently no vaccine available for the H7 subtype of influenza A virus. Virus-specific cellular immune responses play a critical role in virus clearance during influenza infection. In this study, we undertook a side-by-side evaluation of two different adjuvants, *Salmonella typhimurium* flagellin (fliC) and polyethyleneimine (PEI), through intraperitoneal administration to assess their effects on the immunogenicity of the recombinant HA1-2 subunit vaccine of H7N9 influenza. The fusion protein HA1-2-fliC and HA1-2 combined with PEI could induce significantly higher HA1-2-specific IgG and hemagglutination inhibition titers than HA1-2 alone at 12 days post-boost, with superior HA1-2 specific IgG titers in the HA1-2-fliC group compared with the PEI adjuvanted group. The PEI adjuvanted vaccine induced higher IgG1/IgG2a ratio and significantly increased numbers of IFN-γ- and IL-4-producing cells than HA1-2 alone, suggesting a mixed Th1/Th2-type cellular immune response with a Th2 bias. Meanwhile, the HA1-2-fliC induced higher IgG2a and IgG1 levels, which is indicative of a mixed Th1/Th2-type profile. Consistent with this, significant levels, and equal numbers, of IFN-γ- and IL-4-producing cells were detected after HA1-2-fliC vaccination. Moreover, the marked increase in CD69 expression and the proliferative index with the HA1-2-fliC and PEI adjuvanted vaccines indicated that both adjuvanted vaccine candidates effectively induced antigen-specific cellular immune responses. Taken together, our findings indicate that the two adjuvanted vaccine candidates elicit effective and HA1-2-specific humoral and cellular immune responses, offering significant promise for the development of a successful recombinant HA1-2 subunit vaccine for H7N9 influenza.

## Introduction

Avian influenza A (H7N9) virus emerged as a human pathogen in China in spring 2013, and by February 2015, 571 human cases had been reported including 212 deaths [1]. Most cases involved severe respiratory illness, and a mortality rate of approximately 30% was recorded among hospitalized patients [2]. The continued spread of these newly emerged H7N9 viruses among poultry in China, together with the possibility of human-to-human transmission, promoted efforts to develop an effective vaccine [3].

Vaccination is the primary and most effective means of preventing influenza-associated morbidity and mortality [4, 5]. Most current avian vaccines, which are primarily based on the chemically inactivated whole virus [6], have some important drawbacks, such as the risk of partial inactivation of the virus, a change in the immunogenic properties of the virus, and the toxicity of the inactivating agent [7]. The high pathogenicity of H7N9 influenza virus exacerbates these problems [8]. To date, no licensed vaccine exists for H7N9 infection. Thus, it is crucial to develop a vaccination strategy to protect against H7N9 influenza.

Compared with traditional vaccines, subunit vaccines confer equal protection, a higher level of bio-security, and reduced vaccine production times [9]. In our previous study, we reported that HA1-2, the globular head domain (aa 62–284) of the influenza hemagglutinin (HA) expressed in *Escherichia coli*, was an effective antigen for use in an influenza H7N9 subunit vaccine [10].

Along with an effective antigen, a subunit vaccine also requires an adjuvant to enhance the immunogenicity of the antigen [11]. Aluminum salts have been used as vaccine adjuvants for more than 80 years [12]. Aluminum is one of the most common adjuvants used in non-living vaccines, with a successful record of use in human vaccination where it promotes antibody-mediated protective immunity [13]. Toll-like receptor (TLR) agonists are considered potential new candidate adjuvants because they are capable of recognizing pathogen-associated molecular patterns and initiating an immune response. One such example is flagellin, a TLR5 ligand. Song et al. first demonstrated that the globular head of HA fused to the flagellin protein, FljB, of *Salmonella typhimurium* elicited protective immunity to lethal challenge [14]. We verified the adjuvant activity of flagellin, combined with the HA1-2 antigen of H7N9 influenza [10]. Polyethyleneimine (PEI) is an organic polycation used extensively as a gene and DNA vaccine delivery reagent. Recently, it has been reported that PEI has robust systemic adjuvanticity when administered with HIV glycoprotein antigens [15]. Thus, we hypothesized that PEI combined with glycoprotein antigen HA1-2 of H7N9 influenza virus might also confer systemic immune-stimulating activity.

The robust and lasting humoral immune response induced by the resulting fusion protein HA1-2-fliC has been demonstrated by our previous findings. However, the cellular immune response has yet to be investigated, which is likely to be essential for immediate and sustained protective immunity. Therefore, in this study, mice were immunized intraperitoneally with the HA1-2 alone, the fusion protein HA1-2-fliC, or HA1-2 combined with PEI or Alum. These different adjuvant combinations were compared to investigate humoral and cellular immune responses and the balance between Th1 and Th2 immune responses in a C3H/HeJ mouse model, with the aim of developing an effective vaccine against H7N9 infection.

## Materials and Methods

### Mice and ethics statements

Six-week-old female C3H/HeJ mice were purchased from the SLAC Laboratory Animal Co. Ltd., Shanghai, China. All animals were housed in isolators and fed a pathogen-free diet and water in a room at 25°C and a 12 h light-dark cycle. The procedures described in this study were approved by the Committee on the Ethics of Animal Experiments of Yangzhou University, Yangzhou, China.

### Virus used for the hemagglutination inhibition (HAI) antigen

The inactivated avian influenza A/chicken/Jiangsu/CZT4/2013 (H7N9) virus provided by the Animal Infectious Disease Laboratory of Yangzhou University was used as the H7 subtype avian influenza HAI antigen.

### Preparation of vaccine candidates

**Fig 1:**
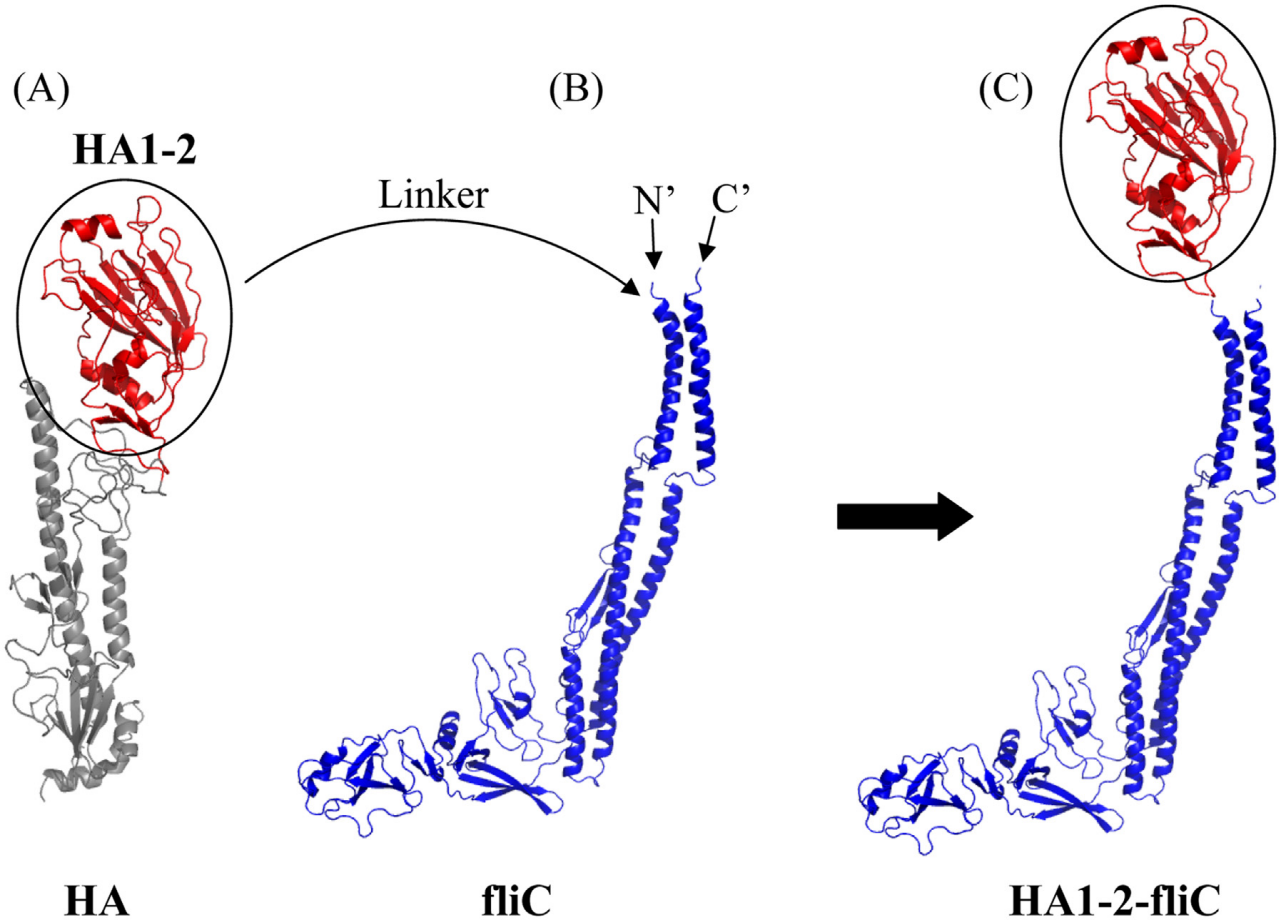
Schematic for generation of the fusion protein HA1-2-fliC. The structural models of the proteins were predicted using the SWISS-MODEL software. Important secondary structural elements, such as α-helixes and β-sheets, are also shown. (A) Ribbon diagram of a monomeric subunit of the HA of avian influenza A (H7N9) virus. The HA1-2 (residues 62–284) of the globular head highlighted in red is circled. (B) The native conformation of *Salmonella typhimurium* fliC. The C- and N- terminals are labeled. (C) HA1-2-fliC construct shown as a ribbon diagram. The globular head of HA1-2 was fused to the N-terminus of fliC by a (Gly_4_Ser)_3_ linker.

The recombinant proteins HA1-2 and HA1-2-fliC (Fig 1) with a His-tag were expressed and purified as described previously [10]. HA1-2-PEI complexes were formed at least 2 h before immunization by adding the antigen into a pre-diluted PEI (branched PEI forms of 25 kD, Sigma-Aldrich, St. Louis, MO, USA) solution followed by immediate mixing. The Alum adjuvant (Thermo Fisher Scientific, Rockford, IL, USA) contained an aqueous solution of aluminum hydroxide and magnesium hydroxide plus inactive stabilizers. Purified HA1-2 protein was mixed with an equal volume of adjuvant (v/v) just prior to immunization.

### Electron microscopy of HA1-2-PEI complexes

For scanning electron microscopy (SEM), 12 μg of PEI was used alone or in combination with 4 μg of HA1-2 in PBS. Samples were prepared as described previously [16]. After sputter coating with gold, the samples were examined using an S-4800 II scanning electron microscope (Hitachi, Tokyo, Japan). For transmission electron microscopy (TEM), samples were prepared according to previously reported methods [17]. In brief, frozen samples were fractured at −120°C in a freeze-fracturing device BAF 060 (Leica Microsystems, Wetzler, Germany), placed onto grids, and viewed on a CM 100 transmission electron microscope (Philips, Amsterdam, The Netherlands).

### Isolation and culture of bone marrow-derived cells (BMDCs)

BMDCs were generated from bone marrow progenitor cells as previously described with some modifications [18, 19]. Briefly, mice were euthanized with an overdose of pentobarbital (60 mg/kg) administered intraperitoneally. The bone marrow cells were obtained from femurs and tibias of female C3H/HeJ mice and cultured in complete RPMI 1640 medium (Thermo Fisher Scientific) supplemented with 10% fetal bovine serum, 1% penicillin-streptomycin, and 10 ng/mL granulocyte-macrophage colony-stimulating factor and interleukin 4 (R&D Systems, Minneapolis, MN, USA) in six-well plates. On days 3 and 5, one third of the total media volume was replaced with fresh complete medium. On day 6, nonadherent and loosely adherent cells were harvested and subcultured overnight in complete medium. On day 7, after washing, only cultures with ≥ 90% of cells expressing CD11c, as determined by flow cytometry (FACS), were used.

### Phenotype analysis of BMDCs

BMDCs were incubated with 2 μg/mL HA1-2 or 2 μg/mL+10 μg/mL HA1-2-PEI complexes for 24 h, then harvested and incubated with biotin-labeled rat anti-mouse CD80 or CD86 (BD Pharmingen, San Diego, CA, USA), respectively, at 4°C for 15 min. After three washes in PBS, the cells were incubated with FITC-labeled streptavidin (BD Pharmingen). After further washing, cells were phenotypically analyzed by FACS analysis.

### Cytokine secretion by BMDCs

BMDCs were incubated with 2 μg/mL HA1-2 or 2 μg/mL+10 μg/mL HA1-2-PEI complexes for 24 h. Then, the levels of IL-6 and IL-12p40 in culture supernatants were measured using mouse enzyme-linked immunosorbent assay (ELISA) kits (BD Biosciences, San Jose, CA, USA) performed according to the manufacturer’s instructions.

### Vaccination and blood sample collection schedule

Groups of C3H/HeJ mice (*n* = 6) were vaccinated intraperitoneally (i.p.) with doses of 10 μg HA1-2, 30 μg HA1-2-fliC (containing 10 μg HA1-2, according to the molecular weight), 10 μg HA1-2 combined with 20 μg PEI, or equal Alum adjuvants (v/v), or 100 μL PBS, on days 0 and 14. The animals were anesthetized with isoflurane and then bled from the retro-orbital sinus with a capillary tube on day 12 after the second immunization. Serum samples were subjected to HAI assays and assays to determine the titers of HA1-2-specific IgG and its subtypes, IgG1 and IgG2a.

### ELISA

Serum titers of antigen-specific IgG, IgG1, and IgG2a were determined by indirect ELISA, as described previously [10]. Briefly, 96-well plates were coated with the 1.5 μg/mL GST-tagged HA1-2 antigen in 50 mM carbonate buffer (pH 9.6) at 4°C overnight and blocked for 2 h at 37°C with blocking buffer (1% bovine serum albumin in PBST). After washing and blocking, serial dilutions of antiserum were added in triplicate and incubated for 2 h at 37°C. Horseradish peroxidase-conjugated goat anti-mouse IgG (1:10000), IgG1 (1:3000), or IgG2a (1:3000) (Invitrogen, Carlsbad, CA, USA) were incubated for 1 h at 37°C as the secondary antibody. 3, 3′, 5, 5′-tetramethybenzidine was used as a substrate to estimate the enzymatic activity. The reaction was stopped with 2 M H_2_SO_4_, and the absorbance was measured at 450 nm using a Microplate Reader (BioTek, Winooski, VT, USA).

### HAI assays

All HAI assays were performed according to the procedure described previously [10]. Briefly, serum samples collected from mice were treated with receptor destroying enzyme II overnight, heat inactivated (56°C, 30 min), diluted in 96-well V-bottomed microtiter plates, and incubated with 4 HA units of inactivated avian influenza A (H7N9) virus for 30 min at room temperature. Then, 1% chicken erythrocytes were added, mixed briefly, and incubated for 30 min at room temperature. The highest dilution of serum that inhibited hemagglutination was considered the HAI titer.

### Isolation of splenic lymphocytes

Two weeks after the booster immunization, all immunized mice were euthanized with an overdose of pentobarbital (60 mg/kg) administered intraperitoneally, and splenic lymphocytes were obtained from the spleens of immunized mice by density gradient centrifugation using Lymphoprep (specific gravity 1.077) (Sigma-Aldrich) as directed by the manufacturer. Single splenocyte suspensions were prepared in complete RPMI 1640 containing 10% fetal bovine serum and 1% penicillin-streptomycin L-glutamine (Gibco, Carlsbad, CA, USA) at a final concentration of 2 × 10^6^ cells/mL.

### Cell activation and FACS analysis

A single-cell suspension of spleen cells was prepared on the 14th day after booster immunization as described above. For HA1-2-specific responses, splenocytes cells were cultured in the presence or absence of the 5 μg/mL HA1-2 antigen for 72 h. Two aliquots of splenocytes (each 5 × 10^5^ cells/100 μL) were incubated for 15 min at room temperature with fluorescently labeled monoclonal antibodies anti-CD4-PE, anti-CD8-APC, or anti-CD69-FITC (BD Pharmingen), and washed three times with PBS. Samples were analyzed using a FACS Aria flow cytometer (BD Biosciences) with FACSDiva software (BD Biosciences).

### Cell proliferation ELISA based on BrdU

Splenic lymphocyte proliferation was evaluated using the commercially available 5-bromo-2ʹ-deoxyuridine (BrdU)-based cell proliferation ELISA kit (Roche Diagnostics, Tokyo, Japan). The assay was performed according to the manufacturer’s protocol. Briefly, 100 μL/well of splenic lymphocytes (2 × 10^5^ cells/well) were pre-treated with or without 10 μg/mL of HA1-2 or ConA (Sigma-Aldrich) in triplicate in 96-well plates and incubated at 37°C, in a 5% CO_2_ incubator for 48 h. Then, the BrdU labeling reagent was added at 10 μL/well. At 12 h, the cells were harvested by centrifugation and the plate was dried at 60°C for 1 h. BrdU-labeled DNA in the cells was fixed and denatured by incubation with FixDenat solution for 30 min at room temperature. After washing, the BrdU-labeled DNA was stained with peroxidase-conjugated anti-BrdU antibody for 90 min at room temperature. The plate was washed again, and TMB substrate solution was added. The reaction was stopped by adding 1 M H_2_SO_4_ solution. Absorbance was measured at 450 nm using a microplate reader (BioTek).

### IFN-γ and IL-4 ELISPOT assay

HA1-2-specific IFN-γ- or IL-4-producing cells were quantified in splenic lymphocyte cultures using the BD^™^ ELISPOT set (BD Biosciences) following the manufacturer’s protocol. Briefly, 96-well ELISPOT PVDF microplates were coated with a capture antibody in sterile PBS, incubated overnight at 4°C, and blocked (2 h at room temperature) with complete RPMI medium. Splenic lymphocytes were counted and plated in ELISPOT plates at 2 × 10^5^ cells/well (in triplicate) in complete RPMI medium. Cells were mock stimulated with RPMI or stimulated with HA1-2 (5 μg/mL) for 24 h incubation at 37°C and 5% CO_2_. After the incubation period, ELISPOT plates were processed following the manufacturer’s specifications using a biotinylated antibody as a detection antibody, streptavidin-AKP (BD Pharmingen) and a BCIP/NBT Liquid Substrate System (Sigma-Aldrich) for assay development (20 min at room temperature). All assay plates were scanned and analyzed using an automated ELISPOT reader system (Bioreader 5000-Vβ, BioSys, Karben, Germany).

### Antigen-specific cytokine assays

Levels of HA1-2-specific IL-6 and IL-23 cytokines were assayed in splenocytes to evaluate cellular immune responses induced by HA1-2-fliC and PEI adjuvanted vaccines. Splenocytes (2 × 10^6^ cells/mL) from immunized mice were incubated for 24 h at 37°C and 5% CO2 in complete RPMI medium with HA1-2 antigen (5 μg/mL). The culture supernatant was collected and concentrations of cytokines were quantitated by ELISA kits for IL-6 (BD Biosciences) and IL-23 (eBioscience), and the IL-6-producing cells were quantified in splenic lymphocyte cultures using the BD™ ELISPOT set (BD Biosciences) according to the manufacturer’s instructions.

### Statistical analysis

All results are expressed as the mean ± standard error of the mean unless otherwise stated. Serum titers of HA1-2-specific IgG, IgG1, and IgG2a were analyzed using log10 transformed data, and HAI serum titers were analyzed using log2 transformed data. The statistical significance between two groups was analyzed using an unpaired Student’s *t*-test with GraphPad Software 5.0 (San Diego, CA, USA). *p* < 0.05 was considered statistically significant.

## Results

### Antigen-PEI complex formation

**Fig 2:**
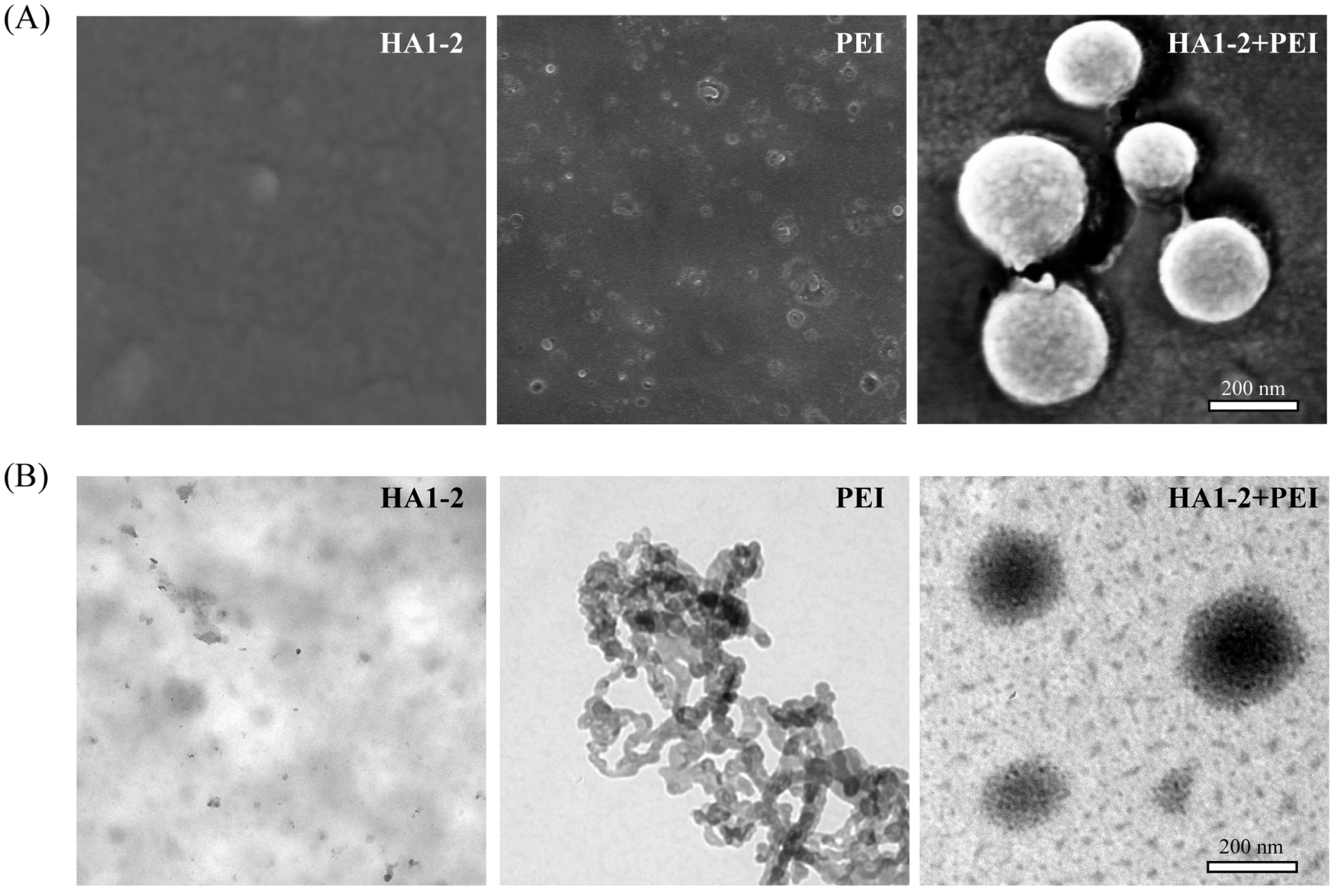
Antigen-PEI complex formation. PEI (12 μg) was used alone or in combination with 4 μg of purified HA1-2 in PBS. (A) HA1-2, PEI, and HA1-2-PEI complexes were observed in randomly chosen fields imaged by SEM. (B) HA1-2, PEI, and HA1-2-PEI complexes were observed in randomly chosen fields imaged by TEM. Scale bar, 200 nm.

We used SEM and TEM to evaluate the HA1-2-PEI complexes. The results showed that PEI alone appeared as an amorphous matrix, whereas HA1-2 and PEI formed a distinct species of particle with an average diameter of 200 nm (Fig 2).

### Adjuvant activity of PEI in BMDCs *in vitro*

**Fig 3:**
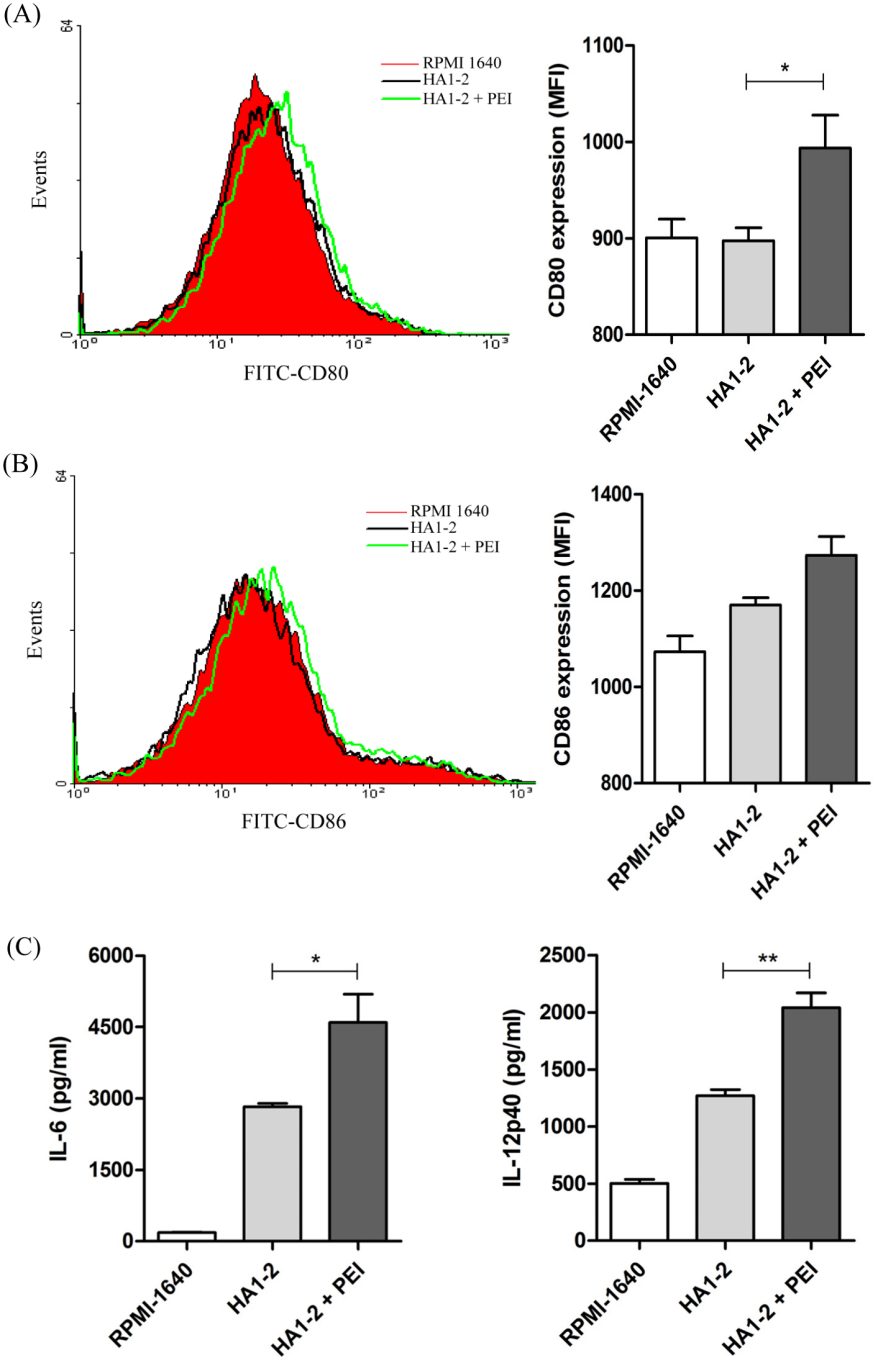
Adjuvant activity of PEI in BMDCs *in vitro*. BMDCs were incubated with the indicated concentrations of HA1-2 or HA1-2-PEI complexes for 24 h. CD80 (A) and CD86 (B) phenotype analysis of BMDCs. The fluorescence intensity of PE-labeled CD80 and CD86 were determined by staining and FACS analysis. (C) Cytokine secretion by BMDCs. Supernatants were collected and tested for IL-6 and IL-12p40 by ELISA. All data are presented as mean ± standard error of the mean; *p < 0.05, **p < 0.01, ***p < 0.001.

To investigate whether PEI modulated BMDC maturation, cellular expression of CD80 and CD86 were analyzed by FACS analysis after exposure to HA1-2 or HA1-2-PEI complexes for 24 h. The results showed that the median fluorescence intensity (MFI) value for CD80 expression stimulated by HA1-2-PEI (993) was significantly higher than that stimulated by HA1-2 (897) (*p* < 0.05). Meanwhile, the MFI value for CD86 expression stimulated by HA1-2-PEI (1273) was also higher than that stimulated by HA1-2 (1169), although with no significant difference (Fig 3A and 3B). The expression levels of CD80 and CD86 were similarly upregulated after exposure to HA1-2-PEI complexes compared with exposure to HA1-2 alone. These data suggested that PEI promoted the HA1-2-induced phenotypic maturation of BMDCs. Dendritic cells are key producers of regulatory and proinflammatory cytokines that play pivotal roles in the regulation of immune responses. To investigate the effects of HA1-2-PEI treatment on cytokine activity, we assessed the secretion of IL-6 and IL-12p40 by ELISA. As expected, the levels of IL-12p40 (2039.7/1269.1 pg/mL) (*p* < 0.01) and IL-6 (4598.4/2825.6 pg/mL) (*p* < 0.05) were significantly increased in the presence of PEI (Fig 3C). These results suggested that PEI promoted cytokine secretion by HA1-2-stimulated BMDCs. Taken together, these findings suggest that PEI assists HA1-2 in enhancing the activation and phenotypic maturation of BMDCs.

### Antibody response to vaccine candidates

**Fig 4:**
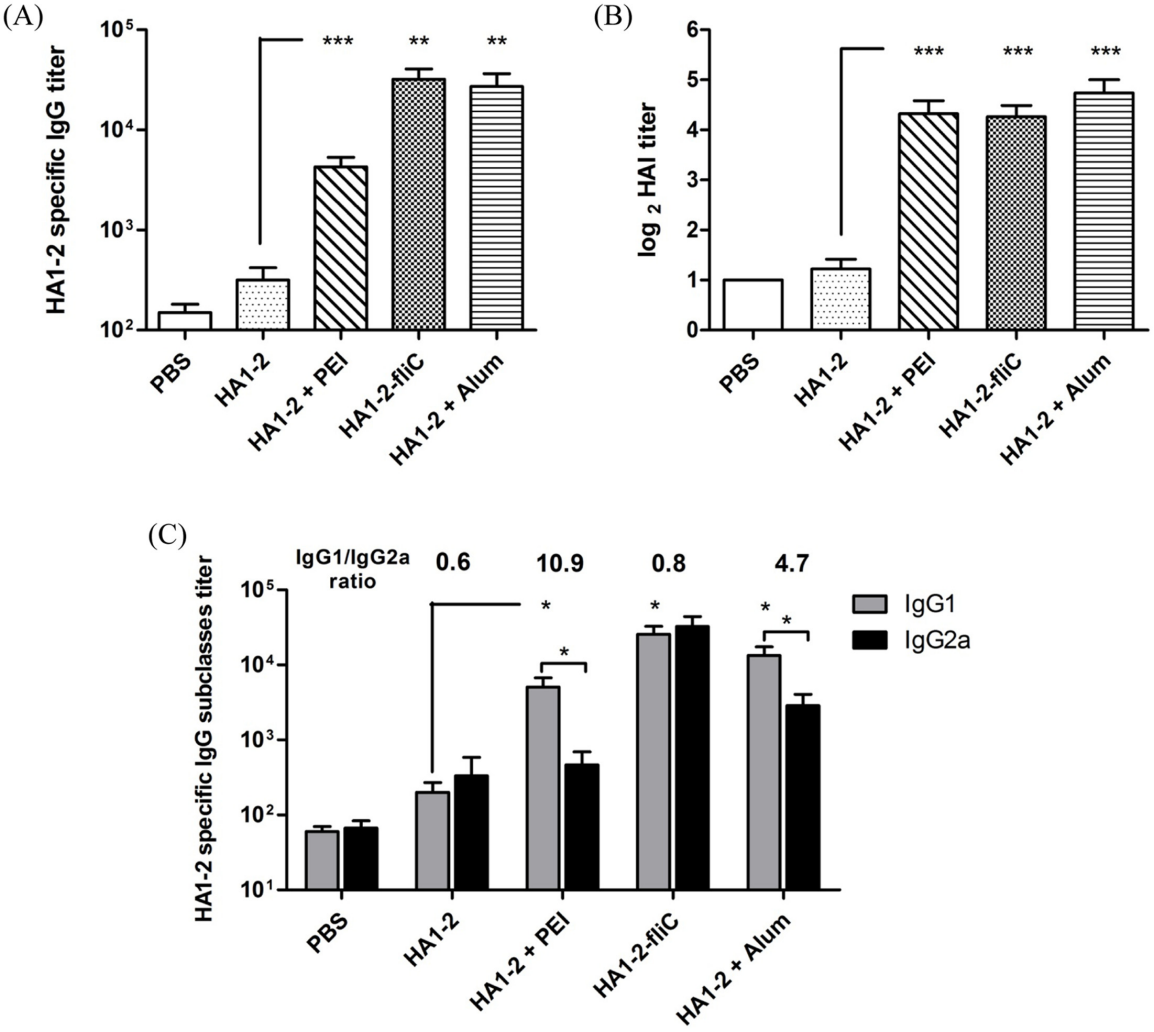
Antibody responses to vaccine candidates. Groups of C3H/HeJ mice (*n* = 6) were vaccinated i.p. with two doses of HA1-2, HA1-2-fliC, PBS, HA1-2 combined with PEI, or Alum on days 0 and 14. The animals were bled 12 days after the second immunization. Antibody titers were measured by ELISA or HAI assays.(A) HA1-2-specific IgG titers. (B) HAI titers. (C) HA1-2-specific IgG subtype (IgG1, IgG2a) titers. All data are presented as mean ± standard error of the mean; *p < 0.05, **p < 0.01, ***p < 0.001.

In this study, mice were immunized (on days 0 and 14) i.p. with HA1-2 alone, HA1-2-fliC, or HA1-2 combined with PEI or Alum. The ability of these vaccine candidates to elicit systemic humoral immunity was assessed by analyzing the serum for HA1-2-specific antibodies (IgG, IgG1, and IgG2a) by ELISA at 12 days post-boost. The results indicated that the adjuvanted vaccines induced significantly higher IgG titers than that induced by HA1-2 alone. Notably, mice immunized with HA1-2-fliC showed the highest levels of IgG, and elicited higher IgG titers than mice immunized with HA1-2-PEI (Fig 4A).

In addition, HAI titers elicited by each of the adjuvant groups were about 8-fold higher than with the HA1-2 antigen alone (20 for PEI adjuvanted vaccine, 19.2 for HA1-2-fliC vaccine, and 2.3 for HA1-2 alone) (Fig 4B). These results indicated that the two adjuvant groups can induce superior immune responses and produce similar titers of HAI, which is consistent with the positive control. There were no statistically significant differences among the adjuvant groups.

### HA1-2-specific IgG subtype response to vaccine candidates

The IgG subtype induced in each group was also evaluated by ELISA. As shown in Fig 4C, IgG1 titers in the groups immunized with PEI and Alum adjuvanted vaccines were significantly higher than the IgG2a titers (*p* < 0.05). The highest IgG1/IgG2a ratio (10.9) was seen with the groups immunized with PEI adjuvanted vaccine, with a lower ratio (4.7) observed with the Alum adjuvanted vaccine. More remarkable, a lower IgG1/IgG2a ratio (0.8) was seen in the HA1-2-fliC group, similar to that seen with HA1-2 alone. Taken together, the IgG1/IgG2a ratios were suggestive of a trend for higher Th2 antibody responses in the PEI adjuvant group, and HA1-2-fliC induced a balanced Th1/Th2 type immune response.

### Cell activation and proliferation

**Fig 5:**
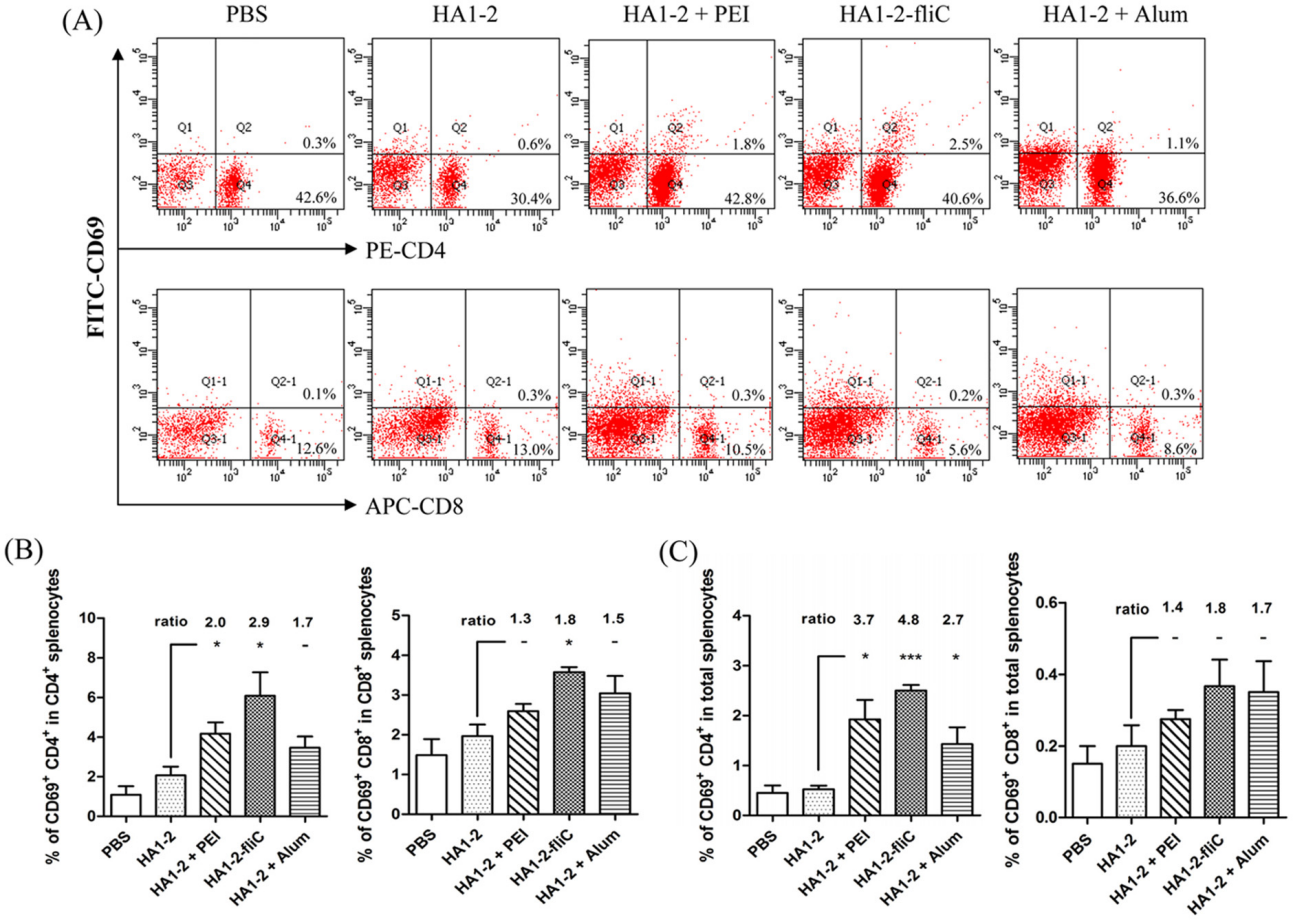
CD69 expression in splenic lymphocytes. At 14 days after the second immunization, splenic lymphocytes of immunized mice were isolated and restimulated with the HA1-2 antigen (5 μg/mL) *in vitro*. The CD69 expression of splenic lymphocytes was determined by FACS analysis. (A) Representative images showing the CD69 expression in splenic lymphocytes. (B) The percentage of CD69^+^ CD4^+^ T cells in CD4^+^ T splenocytes, or CD69^+^ CD8^+^ T cells in CD8^+^ T splenocytes. (C) The percentage of CD69^+^CD4^+^ or CD69^+^CD8^+^ T cells in the total splenocytes. All data are presented as mean ± standard error of the mean; *p < 0.05, **p < 0.01, ***p < 0.001.

**Fig 6:**
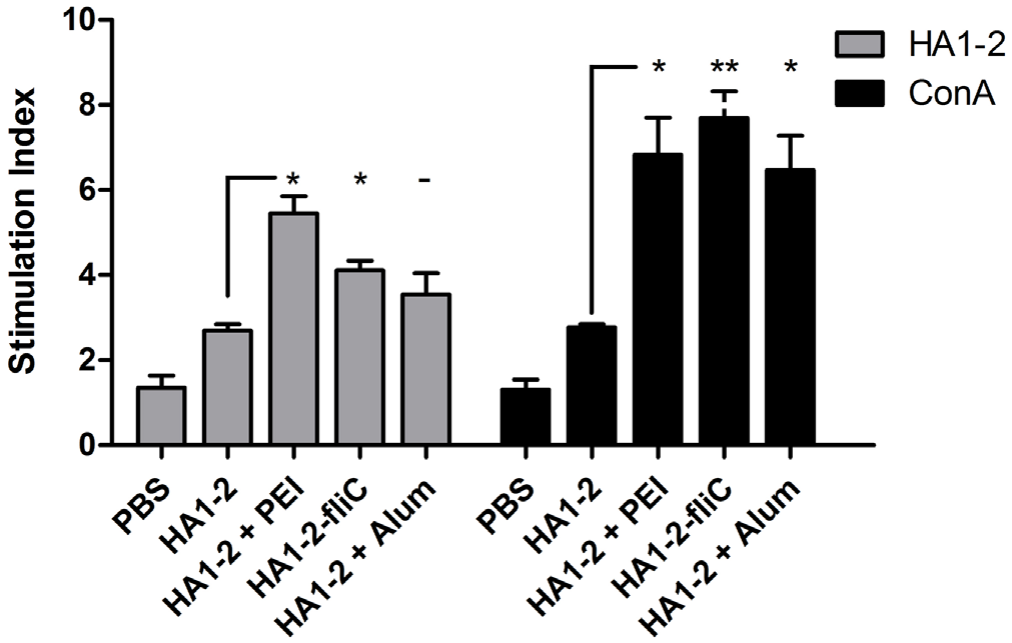
Stimulation index of splenocytes. Splenocytes were prepared from the spleens of mice after the second immunization, then the stimulation index of these cells in response to purified HA1-2 (10 μg/mL) and ConA (10 μg/mL) protein was analyzed using the cell proliferation ELISA assay. The stimulation index (SI) of cell proliferation was calculated using the following equation: SI = (OD450-OD690 of antigen-treated cells)/(OD450-OD690 of untreated cells). All data are presented as mean ± standard error of the mean; *p < 0.05, **p < 0.01, ***p < 0.001.

All immunized mice were euthanized at 14 days after the second vaccination. Splenic lymphocytes were isolated from the immunized mice and were assessed for CD69 activation by FACS analysis. Isolated splenic lymphocytes (2 × 10^5^ cells) were also restimulated with HA1-2 for 48 h, and the proliferative response was detected using BrdU assays according to the manufacturer’s instruction. The percentage of CD69-producing CD4^+^ T cells among the HA1-2 antigen-stimulated CD4^+^ T splenocytes of mice immunized with the PEI adjuvanted vaccine and HA1-2-fliC significantly increased (2.0- and 2.9-fold, respectively) compared with those immunized with HA1-2 alone (*p* < 0.05). Similarly, the percentage of CD69-producing CD8^+^ T cells among the CD8^+^ T splenocytes in the HA1-2-fliC group also significantly increased (1.8-fold on average) (Fig 5B), and a similar trend was observed in the total splenocytes (Fig 5C). The proliferative index of splenocytes stimulated with the HA1-2 antigen was also detected. Compared with the response to HA1-2 alone, HA1-2-fliC and PEI adjuvanted vaccine induced significantly greater specific proliferation of splenic lymphocytes, 1.5-fold and 2.0-fold, respectively (*p* < 0.05), and higher nonspecific proliferation, 2.8-fold (*p* < 0.01) and 2.5-fold (*p* < 0.05), respectively (Fig 6). The percentage of CD69 expression in splenocytes and the proliferative index of splenocytes were markedly increased in the HA1-2-fliC and PEI adjuvanted vaccine groups compared with the HA1-2-alone group. Taken together, these results indicated that two vaccine candidates effectively induced antigen-specific cellular immune responses.

### IFN-γ- and IL-4-producing cells induced by vaccine candidates

**Fig 7:**
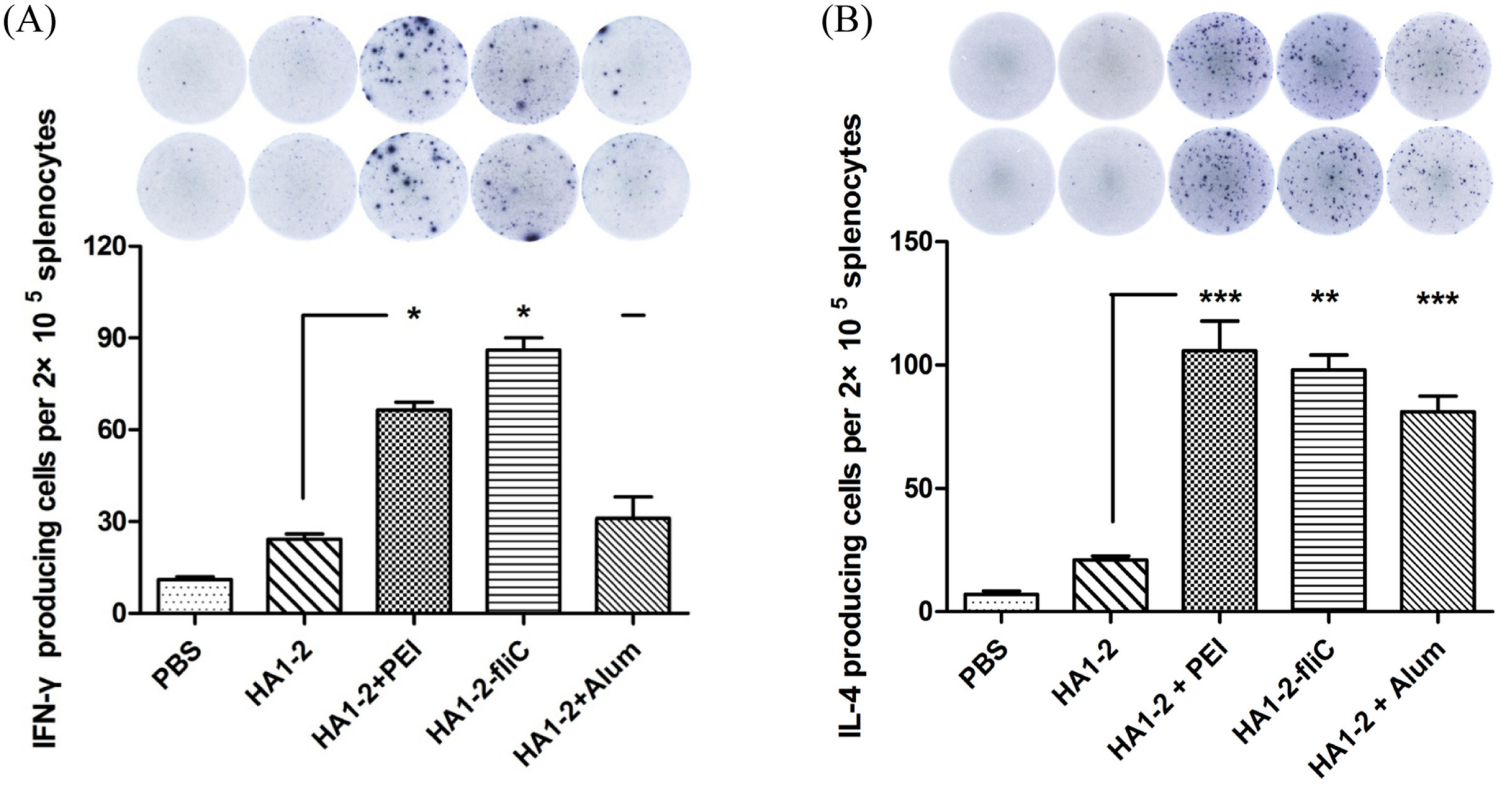
Analysis of IFN-γ and IL-4 by ELISPOT assays. On day 14 after the second immunization, mice were euthanized and single-cell suspensions were prepared from the spleens, cultured for 24 h, and stimulated with purified protein HA1-2 (5 μg/mL). IFN-γ (A) and IL-4 (B) secretion by splenic lymphocytes stimulated with purified HA1-2 was detected by ELISPOT in triplicate wells. All data are presented as mean ± standard error of the mean; *p < 0.05, **p < 0.01, ***p < 0.001.

To further estimate the ability of the vaccine candidates to elicit a specific cellular immune response and to evaluate the immune types, the numbers of IFN-γ- and IL-4-producing cells were determined by ELISPOT assays. Splenocytes were prepared on day 14 after the second vaccination and stimulated with HA1-2 protein (5 μg/mL) *in vitro*. The results indicated that the average number of IFN-γ-producing cells in HA1-2-fliC and PEI adjuvanted vaccination groups (86 and 66, respectively) was significantly higher than in the HA1-2 vaccination group (24) (*p* < 0.05). Similarly, we found that HA1-2-fliC (98) and PEI adjuvanted vaccine (113) induced significantly more IL-4-producing cells than HA1-2 vaccinations (24). Notably, the number of IL-4-producing cells induced in response to vaccination with PEI or Alum adjuvanted vaccine (113 and 74, respectively) was significantly higher than that of IFN-γ-producing cells (66 and 31, respectively) (Fig 7A and 7B), indicating that mixed Th1/Th2 immune responses with a Th2 bias were induced by PEI adjuvanted vaccine. The numbers of IFN-γ- and IL-4-producing cells were essentially equal in the HA1-2-fliC vaccinated group, indicating that both Th1 and Th2 types of immune responses were induced by HA1-2-fliC vaccination.

### Antigen-specific IL-6 and IL-23 cytokine assays

**Fig 8:**
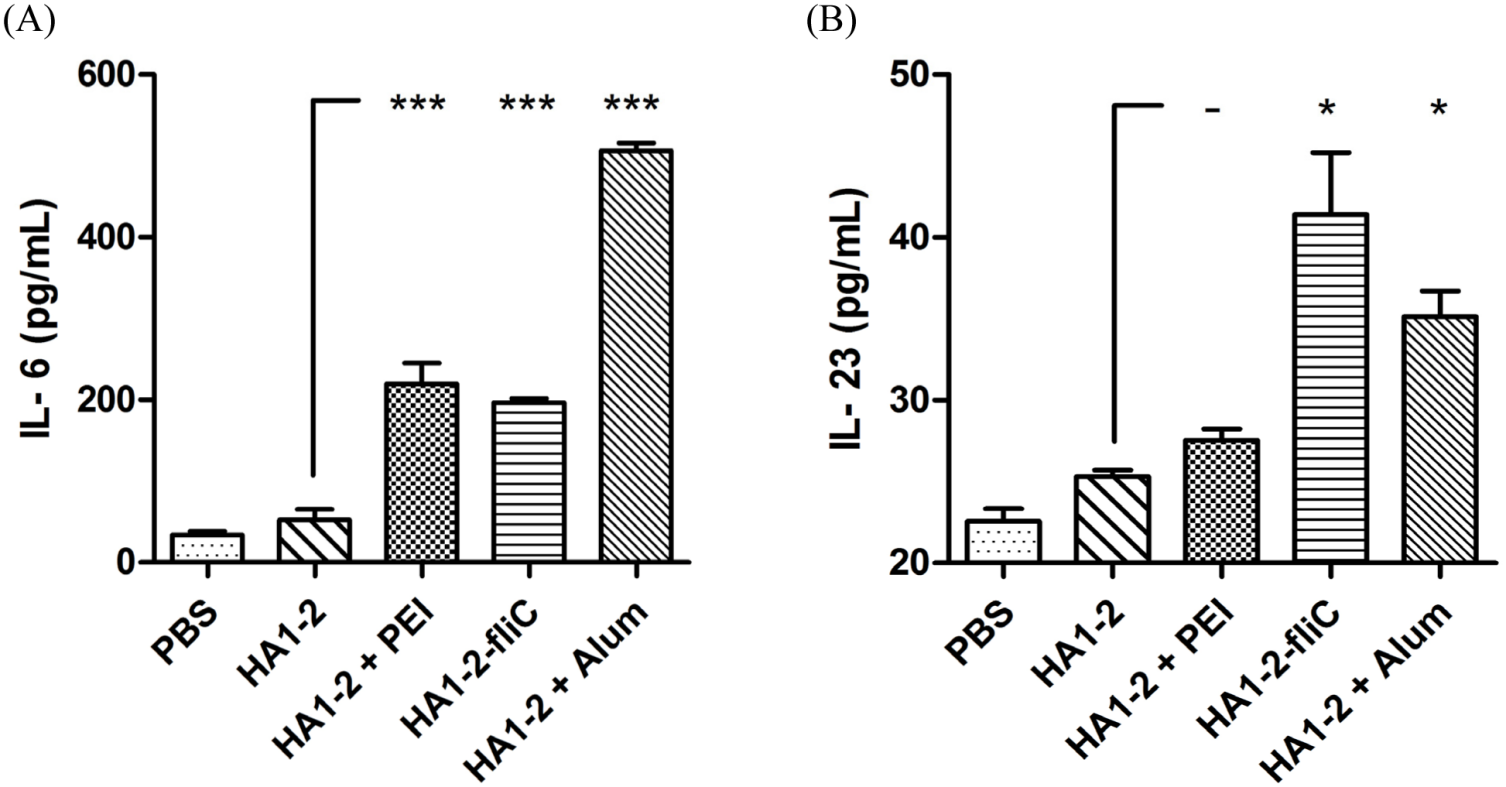
Analysis of antigen-specific cytokine assays. On day 14 after the second immunization, mice were euthanized and single-cell suspensions were prepared from the spleens, cultured for 24 h, and stimulated with purified protein HA1-2 (5 μg/mL). IL-6 (A) and IL-23 (B) cytokines secreted by splenic lymphocytes were detected by commercial ELISA kits in triplicate wells. All data are presented as mean ± standard error of the mean; *p < 0.05, **p < 0.01, ***p < 0.001.

Both IL-6 and IL-23 cytokines are very important in TLR5-flagellin activation and cellular immune response. Thus, we evaluated cytokine production by analyzing the supernatant from splenocytes after HA1-2 antigen stimulation *in vitro*. Two week post-vaccination, we observed significantly increased levels of IL-6 cytokine in all adjuvanted groups by ELISA (*p* < 0.001) and ELISPOT (*p* < 0.01) (Fig 8A and S1 Fig). Moreover, the results also revealed that the production of IL-23 cytokine in HA1-2-fliC vaccinated group was significantly higher than that in HA1-2 group (*p* < 0.05) (Fig 8B).

## Discussion

Recombinant subunit vaccines provide safe and targeted protection against viral infections; however, they are generally less potent than whole viruses. Adjuvants play an important role in the efficacy of vaccines [11]. Both flagellin and PEI adjuvants induce innate immune responses and have shown good immunogenicity in trials, with some receiving approval for influenza immunization [5, 16, 20, 21] and immunization against other viral diseases [22, 23].

Flagellin has been well characterized for its adjuvant activity owing to its TLR5 and Nod-like receptor CARD domain-containing protein 4-binding sites located in a domain also responsible for polymerization [24, 25]. For PEI, the mechanism of innate immune activation is dependent upon the Irf3 IFN response pathway, triggered by activation of intracellular dsDNA sensors. Additionally, PEI reversibly sequesters antigen, recruiting antigen-presenting cells to the site of administration and targeting antigen to antigen-presenting cells [16]. Dendritic cells are a potent type of antigen-presenting cell with the unique ability to induce primary immune responses [26]. When incubation of HA1-2 was combined with PEI on BMDCs, the expression of CD80 was significantly increased compared with HA1-2 alone (*p* < 0.05), and the expression of CD86 was also increased but with no significant difference (Fig 3A and 3B). One possibility is that PEI assists HA1-2 to enter cells. Dendritic cells have the ability to regulate inflammatory responses through promoting the generation of cytokines and chemokines [27, 28]. Mature dendritic cells display antigens initiating specific T-cell immune responses [29] via the production of IL-1β and IL-6. IL-1β is known to induce dendritic cells to produce IL-12 [30, 31]. These findings support our data that the levels of IL-6 and IL-12p40 were significantly increased (Fig 3C).

Previous reports have demonstrated the excellent immunogenicity and efficacy of these vaccine candidates, revealing the physical linkage of HA to flagellin after exposure to influenza in animal models [14, 20]. However, the use of PEI in studies of influenza vaccine is rare. A well-known mechanism for improving the immunogenicity of existing influenza vaccines involves the addition of adjuvants. Another strategy to improve the immunogenicity of inactivated vaccines is to use alternate routes of administration. In this study, we compared PEI and fliC in a more conventional format using prime and booster immunizations to increase adaptive immune responses to the H7N9 subunit vaccines. In our study, HA1-2-specific IgG levels in the serum induced by HA1-2-fliC were higher than those induced by HA1-2 combined with PEI but were equivalent in the HAI titer (Fig 4A and 4B), which were all significantly higher than that of HA1-2 alone, indicating the increased potency of HA1-2 as an immunogen. In similar research reported by Sheppard et al., PEI was shown to elicit robust systemic antigen-specific antibody responses in both subcutaneous and intraperitoneal models of immunization [15].

The development of new adjuvants that enhance both cell- and antibody-mediated immunity is crucial for control of influenza virus infection. Therefore, we wanted to determine whether differences existed in the IgG isotypes produced by different vaccine adjuvants in a C3H/HeJ mouse model, as the IgG1 and IgG2a subtypes have been previously identified as more prominent in Th2 and Th1 responses, respectively [32]. We found that mice received the PEI adjuvant revealed a Th2 skewed response with the highest IgG1/IgG2 ratio, consistent with Alum adjuvant (Fig 4C). The subclass of IgG induced after immunization is an indirect measure of the relative contribution of Th1-type versus Th2-type cytokines [33]. To further estimate the type of immune response and the ability of the vaccine candidates to elicit a cellular immune response, the numbers of IFN-γ- and IL-4-producing cells were determined by ELISPOT assays. Taken together, our findings indicated that PEI induced a Th1 and Th2 mixed immune response with a skew towards a Th2 profile with significantly higher numbers of IL-4 producing cells. We showed that PEI adjuvanted vaccine could elicit a significantly stronger IFN-γ response upon antigen recall than HA1-2 alone, similar to the cytokines released from antigen-restimulated splenocytes of mice immunized with gp140 and PEI, which produced significant amounts of Th1/Th2 cytokines [15]. The levels of cytokines produced by vaccinated animals were analyzed in medium from cultured splenocytes. Splenocytes obtained from mice vaccinated with HA1-2-fliC produced significantly higher levels of IL-6 (*p* < 0.001) and IL-23 (*p* < 0.05) compared with HA1-2 vaccinated group (Fig 8), consistent with the results of the IL-6-producing cells by analyzing the splenic lymphocyte cultures using the ELISPOT assay (S1 Fig), which revealed that both IL-6 and IL-23 cytokines are very important in flagellin-TLR5 activation and cellular immune response [34, 35]. Splenocytes obtained from PEI adjuvanted group produced high level of IL-6 (Fig 8A). Combined with the IL-6 secretion by BMDCs with HA1-2-PEI complexes stimulation *in vitro* (Fig 3C), these results suggested that PEI could promote the secretion of IL-6 to enhance the immune response. Consistent with this, the PEI adjuvanted vaccine elicited markedly increased CD69 expression (Fig 5) and a higher proliferative index of splenic lymphocytes than the HA1-2 antigen alone (Fig 6).

Notably, as previously mentioned, we found that HA1-2-fliC induced not only the highest HA1-2-specific IgG1 antibody responses, but also higher levels of IgG2a than the HA1-2 group. The HA1-2-fliC vaccine also elicited the lowest IgG1/IgG2a ratio in favor of a mixed Th1/Th2 type response in a C3H/HeJ mouse model. Some studies [36, 37] reported that co-delivery of flagellin with an antigen resulted in Th2 responses *in vivo*. We hypothesized that different strains of mice will also affect the immune response bias. For example, the cytokine profile in C3H/HeJ female mice was a mixture of Th1 and Th2 responses, whilst a mainly Th1 profile was observed in C57BL/6 mice after allogeneic and syngeneic vaccination against prostate cancer [38]. In this study, HA1-2-fliC induced both humoral and cellular immune response in C3H/HeJ mice, as determined by IgG1/IgG2a ratios and ELISPOT analysis (Figs 4C and 7).

The mixed Th1/Th2 response induced by PEI is adequate for eliciting high titer antibody responses but is unlikely to be optimal for co-induction of cytotoxic T cells that require a Th1 cytokine environment [16]. It was reported that co-formulation of PEI with the TLR ligand CpG antisense oligodeoxynucleotides synergistically increased the magnitude of the adaptive immune response and biased the response towards Th1 [15]. Combined with our results showing that PEI induced a more Th2-biased immune response (Figs 4C and 7), we hypothesized that the combination of PEI with HA1-2-fliC may synergistically enhance the immunogenicity of the HA1-2 antigen. These consistent signaling interactions both activated innate immune responses, and PEI might be a useful platform for the delivery of TLR5 ligands to their site of action. These combinations may represent promising candidates for immunomodulation and vaccine adjuvant development. A well-known mechanism for improving the immunogenicity of existing influenza vaccines involves the addition of adjuvants. Another strategy to improve the immunogenicity of inactivated vaccines is to use alternate routes of administration [39]. In this regard, the adjuvants PEI and fliC could be optimized for mucosal delivery, to better mimic the natural route of virus infection and potentially elicit mucosal immune responses to H7N9 influenza.

## Conclusions

Our data demonstrate the advantages of two adjuvants, fliC and PEI, for the induction of both T-cell and antibody responses. HA1-2-PEI complexes and HA1-2-fliC enhanced HA1-2 induced immunity, suggesting that the novel HA1-2 combined with PEI or fusion protein HA1-2-fliC represents a promising strategy for H7N9 influenza vaccination.

## Supporting Information

S1 Fig Analysis of IL-6 by ELISPOT assays. On day 14 after the second immunization, mice were euthanized and single-cell suspensions were prepared from the spleens, cultured for 24 h, and stimulated with purified protein HA1-2 (5 μg/mL). IL-6 secretion by splenic lymphocytes was detected by ELISPOT in triplicate wells. All data are presented as mean ± standard error of the mean; *p < 0.05, **p < 0.01, ***p < 0.001.
(PDF)
