# Supplementary material for: Immunopotentiation of Different Adjuvants on Humoral and Cellular Immune Responses Induced by HA1-2 Subunit Vaccines of H7N9 Influenza in Mice
Source: PLoS One. 2016 Mar 1;11(3):e0150678. doi: 10.1371/journal.pone.0150678 (PMC4773109; doi:10.1371/journal.pone.0150678)
Supplement: S1 Fig — On day 14 after the second immunization, mice were euthanized and single-cell suspensions were prepared from the spleens, cultured for 24 h, and stimulated with purified protein HA1-2 (5 μg/mL). IL-6 secretion by splenic lymphocytes was detected by ELISPOT in triplicate wells. All data are presented as mean ± standard error of the mean; *p < 0.05, **p < 0.01, ***p < 0.001. (PDF) [file pone.0150678.s001.pdf]

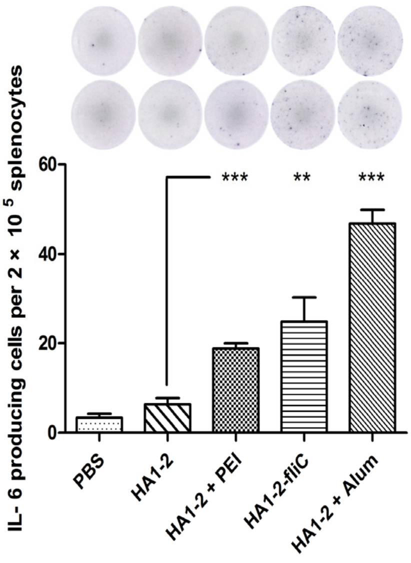

S1 Fig. Analysis of IL-6 by ELISPOT assays. On day 14 after the second immunization, mice were euthanized and single-cell suspensions were prepared from the spleens, cultured for 24 h, and stimulated with purified protein HA1-2 (5 μg/mL). IL-6 secretion by splenic lymphocytes was detected by ELISPOT in triplicate wells. All data are presented as mean ± standard error of the mean; **p* < 0.05, ***p* < 0.01, ****p* < 0.001.
